# We can also do online – evaluation of the accompanying digital seminar of the elective subject “General Practice” during intership (PJ) at Ruhr-University Bochum

**DOI:** 10.3205/zma001469

**Published:** 2021-04-15

**Authors:** Irmgard Streitlein-Böhme, Barbara Woestmann, Horst Christian Vollmar, Klaus Böhme

**Affiliations:** 1 Ruhr University Bochum, Medical Faculty, Institute of General Practice and Familiy Medicine, Bochum, Germany

**Keywords:** digital teaching, internship, elective subject general practice, seminar program

## Abstract

**Aim: **Due to the lockdown caused by the Corona pandemic, the internship (PJ=practical year) seminars of the elective subject “General Practice” at Ruhr-University Bochum had to be transferred on short notice into online teaching formats via a digital platform. At the end of these new online course, the four students evaluated the comprising 16 teaching units.

**Methods: **The seminars, each comprising four teaching units, took place in online blended-learning units and online events. After completing the seminar program, the four participants filled out a written questionnaire regarding the implementation of digital teaching, quality of teaching content, acceptance as well as advantages and disadvantages of the teaching format.

**Results:** The acceptance of digital teaching was very high among students. Advantages and disadvantages of this teaching format compared to the previous face-to-face events became apparent; a positive assessment of the possibilities of the online format clearly prevailed, as competence-oriented, interactive aspects were very well implemented.

**Conclusion:** Due to the need of switching to digital teachings formats, new, innovative perspectives have arisen for PJ teaching in Bochum as well as for the more distant second location Ostwestfalen-Lippe. This is particularly true with regard to centralised seminar offers despite decentralised training centres. When implementing “new” licensing regulations this creates an opportunity for general practice to include teaching practices in training throughout the country.

## Zusammenfassung

**Zielsetzung: **Durch den coronabedingten Lockdown mussten die PJ-Seminare im Rahmen des Wahlfaches Allgemeinmedizin an der Ruhr-Universität Bochum kurzfristig über eine digitale Plattform in entsprechende Online-Lehrformate überführt werden. Am Ende wurde diese insgesamt 16 Unterrichteinheiten (UE) umfassende Online-Veranstaltung von den im PJ-Tertial befindlichen 4 Studierenden evaluiert.

**Methode: **Die jeweils 4 UE umfassenden Seminare fanden im Online-Format in Form von Blended-Learning-Einheiten sowie reinen Online-Präsenzveranstaltungen durchgeführt. Nach Abschluss des Seminarprogramms erfolgte durch die 4 Teilnehmer*innen (TN) des PJ-Tertials eine schriftliche Evaluation mit Fragen zur Umsetzung der digitalen Lehre, zur Qualität der Lehrinhalte sowie zu Akzeptanz und zu Vor- und Nachteilen des Lehrformates.

**Ergebnisse: **Die Akzeptanz der digitalen Lehre war bei den Studierenden sehr hoch. Es zeigten sich Vor- und Nachteile dieses Lehrformates gegenüber den bisherigen Präsenzveranstaltungen, wobei aber eine positive Bewertung der Möglichkeiten des Online-Formates deutlich überwog, da auch kompetenzorientierte, interaktive Aspekte sehr gut umgesetzt werden konnten.

**Schlussfolgerung: **Durch die Notwendigkeit auf digitale Lehrformate umzustellen, haben sich für die PJ-Lehre am Standort Bochum sowie für den weiter entfernten Zweitstandort Ostwestfalen-Lippe neue, innovative Perspektiven ergeben. Dies gilt besonders auch im Hinblick auf zentrale Seminarangebote trotz dezentraler Ausbildungsstätten. Dadurch ergibt sich für die Allgemeinmedizin die Möglichkeit, bei der Umsetzung einer neuen Ärztlichen Approbationsordnung auch bundesweit PJ-Praxen in die Ausbildung mit einzubinden.

## Introduction

During their internship (PJ) tertial in General Practice, medical students had the opportunity to visit seminars which were held in four-week intervals at the campus. After the Corona lockdown in March 2020 [https://recht.nrw.de/lmi/owa/br_vbl_detail_text?anw_nr=6&vd_id=18406] these seminars had to be changed at short notice to an online format via a digital platform for the second tertial of the November 2019/20 intership cohort.

## Project description

Between April and June 2020 four seminars of four teaching units were conducted online; the content based on the previous face-to-face events:

- two case seminars on common chronic diseases and typical acute consultation in general practice;
- one seminar for practising ECG analysis and visual diagnostics of dermatological clinical pictures in general practice;
- one seminar on injuries in general practice and mock examinations in preparation for the M3 examination.

The students received preparatory tasks in form of patient cases or other materials (e.g. ECG printouts) before the events. Besides this blended-learning format, students received materials for joint processing and subsequent presentation during the seminar. After completing all seminars the students (n=4) were interviewed about their experiences with the digital course. Therefore, they filled out a questionnaire with 29 items addressing digital teaching and the quality of the single teaching content. In addition, free-text questions were asked: what the students particularly liked, which areas should be optimised and which advantages and disadvantages of the digital teaching offer were seen. For questions regarding digital teaching, an already developed questionnaire from the Medical Faculty of Freiburg was used (tested in a pilot project). This questionnaire has not been published yet.

## Results

The survey on digital teaching (e.g., the digital learning content promoted my understanding of the topic/the structure of the digital learning content was easy for me to follow) and the quality of teaching content resulted in good to very good ratings. The results are not presented in a differentiated way due to the limited validity of four participants.

The following exemplary free-text comments resulted:

What did you like in particular?

- “The seminars were always set up interactively and not in a frontal lecture. This gave me the feeling that we were learning in a group, even though you were sitting alone in front of the PC. I also liked the embedded group work.”
- “I particularly liked the ECG and dermatology exercises."
- “I also liked that we talked about diagnostic methods despite the setting and that we, for example watched a video on the case of non-specific lower back pain.”
- “Overall, I thought it was very good, that it was possible to offer such a comprehensive and well-structured seminar series “despite Corona” and that it has not been cancelled like in some hospitals. For me personally, a certain amount of theoretical support/follow-up in the PJ was very helpful. But I also liked the fact that it wasn't too much theory.”

An advantage of digital teaching was seen in the reduction of travel time (several hours for some of the participants) to the university. 

The following disadvantages were mentioned:

- “Learning together in a face-to-face class is quite different from sitting alone in front of your PC. But this is certainly also a matter of habits.”
- “We would have had the opportunity in a non-digital seminar to have diagnostic techniques demonstrated and then practice them on each other.”

## Discussion

This report is based on a complete evaluation of all participants of the internship (PJ) tertial “General Practice” from March to July 2020. Due to the small number of students, the validity is limited.

The digital seminars showed a high acceptance among students, as Kuhn et al. mentioned in 2018 [1], whereby both advantages and disadvantages were seen. The advantages were clearly the flexibility and time saved by meeting in the digital space. The disadvantages were that diagnostic techniques could not be practised or professionalised in person. 

The results show, that a variety of seminar contents can be taught online just as well as in face-to-face events. This is especially true for ECG analysis and dermatological clinical pictures. There is the possibility of using image and film materials to practice conversation techniques or discuss case reviews. Thus, frontal and interactive teaching formats are possible side by side in small groups. Group work in particular is easy to carry out with the digital media and in “Corona times”, an extremely useful alternative to small group work in a classroom setting. With this media, the implementation of mock examinations in competence-based form is excellently realisable.

## Conclusion

For the execution of the internship (PJ) elective subject “General Practice” in our more distant secondary location Ostwestfalen-Lippe and other decentralised PJ teaching practices have arisen new, interesting perspectives in relation to a digital seminar offer for PJ students. This applies especially for the implementation of the “new” licensing regulations [2]. A more flexible nationwide choice of internship (PJ) practice would be possible, with simultaneous seminar participation to one's own location and faculty-specific preparation for the final M3 examination. If the final examination (M3) becomes mandatory in the subject “General Practice”, even those students who do not want to complete a PJ quarter (instead of tertial) in general practice can experience good preparation with digital learning opportunities for their final M3 examination.

## Competing interests

The authors declare that they have no competing interests. 
